# Decomposing urban-rural differences in multimorbidity among older adults in India: a study based on LASI data

**DOI:** 10.1186/s12889-022-12878-7

**Published:** 2022-03-15

**Authors:** Shekhar Chauhan, Shobhit Srivastava, Pradeep Kumar, Ratna Patel

**Affiliations:** 1 Department of Family and Generations, International Institute for Population Sciences, Mumbai, India; 2 Research Scholar, International Institute for Population Sciences, Mumbai, India; 3 Consultant- Research & Data Analysis, Population Council India Office, Zone 5A, India Habitat Centre, Lodi Road, 110003 New Delhi, India; 4 Department of Public Health and Mortality Studies, International Institute for Population Sciences, Mumbai, India

**Keywords:** Multimorbidity, Urban-rural differences, Obesity, LASI, India

## Abstract

**Background:**

Multimorbidity is defined as the co-occurrence of two or more than two diseases in the same person. With rising longevity, multimorbidity has become a prominent concern among the older population. Evidence from both developed and developing countries shows that older people are at much higher risk of multimorbidity; however, urban-rural differential remained scarce. Therefore, this study examines urban-rural differential in multimorbidity among older adults by decomposing the risk factors of multimorbidity and identifying the covariates that contributed to the change in multimorbidity.

**Methods:**

The study utilized information from 31,464 older adults (rural-20,725 and urban-10,739) aged 60 years and above from the recent release cross-sectional data of the Longitudinal Ageing Study in India (LASI). Descriptive, bivariate, and multivariate decomposition analysis techniques were used.

**Results:**

Overall, significant urban-rural differences were found in the prevalence of multimorbidity among older adults (difference: 16.3; *p* < 0.001). The multivariate decomposition analysis revealed that about 51% of the overall differences (urban-rural) in the prevalence of multimorbidity among older adults was due to compositional characteristics (endowments). In contrast, the remaining 49% was due to the difference in the effect of characteristics (Coefficient). Moreover, obese/overweight and high-risk waist circumference were found to narrow the difference in the prevalence of multimorbidity among older adults between urban and rural areas by 8% and 9.1%, respectively. Work status and education were found to reduce the urban-rural gap in the prevalence of multimorbidity among older adults by 8% and 6%, respectively.

**Conclusions:**

There is a need to substantially increase the public sector investment in healthcare to address the multimorbidity among older adults, more so in urban areas, without compromising the needs of older adults in rural areas.

## Background

Declining fertility rates and increasing life expectancy have increased the population of older adults worldwide [1]. As per the World Population Prospects report, by 2050, 1 in 6 people worldwide would be over 65 years of age, up from 1 to 11 in 2019 [2]. Nearly all the societies in the world are in the midst of this longevity revolution, albeit at a different stage with differing pace [2]. Like other developing countries, India is also in the transition phase, experiencing an increase in the proportion of older adults’ population [3]. Considering increasing education and improving health facilities, the share of the older adults’ population (60+ years) in India has increased from 5.3% to 1971 to 5.7% in 1981 and further from 6% to 1991 to almost 8% in 2011 [4]. Furthermore, it is evident that older adults’ proportion in India will continue to increase in the future, however, concerning various health problems [5]. While global ageing depicts a triumph of medical, social, and economic advances over disease, it also represents tremendous challenges [6]. Multimorbidity is one such challenge that becomes very prominent during ageing [7]. Developed as well as developing countries [8–11] including India [12, 13] are experiencing a rise in the prevalence of multimorbidity among older adults.

Multimorbidity is defined as the co-occurrence of two or more than two diseases in the same person [14].With rising longevity, multimorbidity has become a prominent concern among the older population. Evidence from both developed and developing countries shows that older people are at much higher risk of multimorbidity [9, 15–17]. Multimorbidity has been associated with several adverse health outcomes among older adults, including reduced physical and cognitive functions [18–23], reduced quality of life [24, 25], elevated risk of death [22, 25], disability [25], and poor functional status [25]. Place of residence (urban-rural differential) is a significant risk factor in the occurrence of multimorbidity among older adults [26]. This becomes even more poignant and alarming considering the higher prevalence of multimorbidity among older adults urban than their rural counterparts [12].

Several studies have examined the prevalence and determinants of multimorbidity among older adults in developed countries [11, 27, 28]; however, the available literature on multimorbidity in developing countries [9, 29, 30] including India [31] is limited. Moreover, almost all of the latest research on multimorbidity among older adults in India is based in community-setting, thereby devoid of any national representation [32–37]. To add more to the literature gap, previously available literature significantly proposed various risk factors of multimorbidity among older adults [31, 32, 34, 35, 38]; however, none of the study exclusively examined rural-urban differential in multimorbidity among older adults in India in recent times utilizing information on a nationally representative large scale survey data. A study examined urban-rural differential in multimorbidity among adults in India [26]. Therefore, this study intends to examine rural-urban differential in multimorbidity among older adults by utilizing data from Longitudinal Ageing Survey in India (LASI), 2017-18. Moreover, this study decomposes the risk factors of multimorbidity and identifies the covariates that contributed to the change in multimorbidity by rural and urban residents.

## Methods

### Data

The study used data from the first wave of the Longitudinal Ageing Study in India (LASI), conducted in 2017-18 [39]. LASI is a national representative survey that gather the economic, health, and social drivers of population ageing in India [39]. About 72,000 older persons in India’s states and union territories were surveyed in LASI [39]. The primary objective of the survey was to look into the physical and social, and economic well-being of older persons in India. To arrive at the final units of observation, LASI used a multistage stratified area probability cluster sampling method [39]. This included older people aged 45 and up, as well as their spouses of any age. The survey used a three-stage sampling in rural areas, whereas in urban areas, it used a four-stage sampling approach [39]. The initial stage in each state/Union Territory (UT) was to choose Primary Sampling Units (PSUs), or sub-districts (Tehsils/Talukas). In the selected PSUs, the second stage entails choosing villages in rural regions and wards in urban areas [39]. In the third step, families were chosen from various communities in rural areas. In rural areas, a fixed number of HHs (i.e. [32]) from each selected village or village segment (for villages with more than 500 HHs) were selected. Sampling in urban areas, on the other hand, required an extra step. In the third step, one Census Enumeration Block (CEB) was chosen randomly in each urban area [39]. Households from this CEB were chosen in the fourth stage. In urban areas, a fixed number of HHs (i.e. [35]) were selected from each selected CEB. Details about survey design and data collection procedure has been published elsewhere [39]. The overall HH response rate was 96% (93% for urban and 97% for rural), and the overall individual response rate was 87% (89.6% for rural and 83.6% for urban). The informed consent was taken before the interview the interview [39]. The Indian Council of Medical Research (ICMR) extended the necessary guidance and ethical approval for conducting the LASI [39]. The present study used data on the eligible respondents age 60 years and above [39]. The total sample size for the present study is 31,464 older adults aged 60 years and above (rural-20,725 and urban-10,739).

### Variable description

#### Outcome description

The outcome variable was categorized as binary, i.e., multimorbidity (no/yes) [40]. Multi-morbidity is defined as the presence of two or more chronic diseases, such as hypertension, stroke, chronic heart disease, diabetes, neurological/psychiatric disease, cancer or malignant tumour, any bone/joint disease, any chronic lung disease, or high cholesterol [39]. The diseases were self-reported, however, diagnosed [41] as was assessed through the question “Has any health professional ever diagnosed you with the following chronic conditions or diseases?” [40].

### Explanatory variables

#### Group variable

The place of residence was categorized as rural and urban. However, an earlier multi-country study stated that urban-rural differences in multimorbidity vary from country to country, and it is suggested to undertake country-wise studies examining urban-rural differences in multimorbidity to predict the necessary strategies to address the multimorbidity [42]. Therefore, this study intends to examine urban-rural differential in multimorbidity among older adults in India.

### Main explanatory variables

#### Obesity-related factors

Overweight/obesity was categorized as no and yes [43]. Obese/overweight was defined as having a body mass index of ≥25 kg/m^2^. No and yes were used to categorise high-risk waist circumference [43]. High-risk waist circumferences were defined as male and female waist circumferences of greater than 102 cm and 88 cm, respectively [44]. No and yes were used to categorise high-risk waist-hip ratios. Males and females with waist-hip ratios more than 0.90 cm and 0.85 cm, respectively, were classified as having a high-risk waist-hip ratio [44].

#### Behavioural factors

Physical activity levels were classified as frequent (every day), rare (once a week, once a month, one to three times a month), and never [40]. Tobacco and alcohol consumption was recoded as no and yes [40]. The tobacco and alcohol consumption were assessed using the question that “did the respondent ever consumed any tobacco related product?” and “did the respondent ever consumed alcohol?”

#### Individual factors

Age was recoded as young old (60-69 years), old-old (70-79 years), and oldest-old (80+ years) [45]. Sex was recoded as male and female. Education was categorized as no education/primary schooling not completed, primary completed, secondary completed, and higher and above. Marital status was coded as currently married, widowed, and others (separated/never married/divorced) [45]. Finally, working status was recoded as working, retired, and not working [45].

#### Household factors

The monthly per capita expenditure (MPCE) quintile was calculated using household consumption data. The sample houses were questioned on food and non-food expenses with sets of 11 and 29 questions, respectively. Non-food expenditure was collected over 30-day and 365-day reference periods, whereas food expenditure was collected over a seven-day reference period. Food and non-food expenses were standardised using a 30-day reference period [39]. As a summary measure of consumption, the monthly per capita consumption expenditure (MPCE) is computed and used. The variable was then divided into five quintiles, i.e., from poorest to richest. Religion was coded as Hindu, Muslim, Christian, and Others. Caste was categorized as Scheduled Tribe (ST), Scheduled Caste (SC), Other Backward Class (OBC), and others. As a result of their low caste status in Hindu society, the Scheduled Caste is a group of people who are socially isolated and financially/economically disadvantaged [40, 46]. The Scheduled Castes and Tribes of India are among the poorest socioeconomic groupings in the country [40, 46]. The OBC is the group of people identified as “educationally, economically and socially backward [40, 46].” The OBCs are considered lower castes in the traditional caste system [40, 46]. The “other” caste category is identified as having higher social status. The geographical region was recodedas North, Central, East, Northeast, West, and South [47].

## Statistical approach

To show the preliminary findings, descriptive analysis and bivariate analysis were used. To analyse the residential differentials and determine the significance level, the proportion test was utilised [48]. In addition, a multivariate decomposition logistic regression analysis was used to identify the contributions of covariates which explain the group differences to average predictions [49]. The aim of the decomposition analysis was to identify covariates that contributed to the change in multimorbidity by rural and urban places of residence.

The compositional differences (endowments) ‘E’ and the effects of characteristics, which are the differences in the coefficients or behavioural change ‘C’ responses for the selected predictor variables, are the two contributing effects in the multivariate decomposition analysis [50]. As a result, the observed variations in multimorbidity may be decomposed additively into characteristics (or endowments) and a coefficient (or effects of features) component [51]. In the non-linear model, the dependent variable is a function of a linear combination of predictors and regression coefficients:

$Y=F\ \left( X\beta \right)= logit\ (Y)= X\beta$, where Y denotes the n*1 dependent variable vector, X an n*K matrix of independent variables, and $\beta$ a K*1 vector of coefficients.

The proportion difference in Y between urban A and urban B of multimorbidity can be decomposed as:

$${Y}_{A}-{Y}_{B}=F\left({X}_{A}{\beta }_{A}\right)-F\left({X}_{B}{\beta }_{B}\right)$$

For the log odds of multimorbidity, the proportion of the model is written as

$$ Logit\left(Y_A\right)-logit\left(Y_B\right)=F\left(X_A\beta_A\right)-F\left(X_B\beta_B\right)=\underbrace{F\left(X_A\beta_A\right)-F\left(X_B\beta_A\right)}_{\mathrm E}+\underbrace{F\left(X_B\beta_A\right)-F\left(X_B\beta_B\right)}_{\mathrm C}$$

The difference due to endowment change is the component ‘E,‘ also known as the explained component. The difference attributed to coefficient (behavioural) change, often known as the unexplained component, is the ‘C’ component.

The model structure for the decomposition analysis was:

$$Logit \left(A\right)-Logit \left(B\right)=\left[{\beta }_{0 A}-{\beta }_{0B}\right]+\sum {\beta }_{ijA}\left[{X}_{ijA}-{X}_{ijB}\right]+\sum {X}_{ijB}\left[{\beta }_{ijA}-{\beta }_{ijB}\right]$$, where

- ${\beta }_{0 A}$ is the intercept in the regression equation for rural
- ${\beta }_{0B}$ is the intercept in the regression equation for urban
- ${\beta }_{ijA}$ is the coefficient of the ${j}^{th}$ category of the ${i}^{th}$determinant for rural
- ${\beta }_{ijB}$ is the coefficient of the ${j}^{th}$category of the ${i}^{th}$determinant for urban
- ${X}_{ijA}$ is the proportion of the ${j}^{th}$category of the ${i}^{th }$determinant for rural
- ${X}_{ijB}$ is the proportion of the ${j}^{th}$category of the ${i}^{th}$determinant for urban

The command *mvdcmp* was used to carry out multivariate decomposition analysis in STATA 14 [52].

## Results

### Socio-economic profile of study population, 2017-18 (Table 1)

The LASI survey covered a panel sample of 72,250 individuals age 45 and above and their spouses. However, this study utilized sample of 31,464 elderly aged 60 and above. Mean age of respondent was 69 years; S.E: 0.042 (for rural-69; S.E: 0.053 and urban-69 years; SE: 0.070). A total of 68% of respondent belonged to rural areas and rest of them were from urban areas (32%). The prevalence of obesity-related factors such as obese/overweight, high-risk waist circumference, and high-risk waist-hip ratio was higher among urban resident older adults than rural counterparts (see Table 1). Moreover, among behavioural factors, rural resident older adults did more frequent physical activity than urban ones (19.2% vs. 15%). Similarly, tobacco (45.2% vs. 26.6%) and alcohol consumption (15.7% vs. 11.3%) was more prevalent among older adults who lived in rural areas than those who lived in urban areas. A higher proportion of older adults belonged to the young-old cohort in both rural and urban areas. The proportion of older adults with no education/primary not completed was higher in rural (77.1%) areas than in urban (46%) areas. Similarly, the percentage of working older adults was higher in rural areas (35.4%) than urban (19.6%) counterparts.

**Table 1:** Socio-economic profile of older adults in India, 2017-18

Background characteristics	Rural		Urban	
	Sample	Percentage	Sample	Percentage
**Obesity related factors**				
** Obese/overweight**				
No	17,863	86.2	7,160	66.7
Yes	2,862	13.8	3,579	33.3
** High risk waist circumference**				
No	17,536	84.6	7,069	65.8
Yes	3,189	15.4	3,670	34.2
** High risk waist-hip ratio**				
No	6,994	33.8	3,016	28.1
Yes	13,731	66.3	7,723	71.9
**Behavioural factors**				
** Physical activity status**				
Frequent	3,980	19.2	1,610	15.0
Rare	3,101	15.0	813	7.6
Never	13,644	65.8	8,317	77.4
** Tobacco consumption**				
No	11,353	54.8	7,886	73.4
Yes	9,372	45.2	2,853	26.6
** Alcohol consumption**				
No	17,465	84.3	9,523	88.7
Yes	3,260	15.7	1,216	11.3
**Individual factors**				
** Age**				
Young-old (60-69 years)	12,139	58.6	6,268	58.4
Old-old (70-79 years)	6,169	29.8	3,354	31.2
Oldest-old (80+ years)	2,417	11.7	1,117	10.4
** Sex**				
Male	10,045	48.5	4,835	45.0
Female	10,680	51.5	5,904	55.0
** Education**				
Not educated/primary not completed	15,986	77.1	4,937	46.0
Primary	2,069	10.0	1,511	14.1
Secondary	1,988	9.6	2,598	24.2
Higher	682	3.3	1,693	15.8
** Marital status**				
Currently married	13,017	62.8	6,315	58.8
Widowed	7,280	35.1	4,162	38.8
Others	427	2.1	262	2.4
** Working status**				
Working	7,341	35.4	2,106	19.6
Retired	8,774	42.3	4,719	43.9
Not working	4,610	22.2	3,913	36.4
**Household factors**				
** MPCE quintile**				
Poorest	4,446	21.5	2,396	22.3
Poorer	4,608	22.2	2,197	20.5
Middle	4,375	21.1	2,207	20.6
Richer	3,932	19.0	2,117	19.7
Richest	3,364	16.2	1,822	17.0
** Religion**				
Hindu	17,309	83.5	8,497	79.1
Muslim	2,021	9.8	1,604	14.9
Christian	623	3.0	269	2.5
Others	772	3.7	369	3.4
** Caste**				
Scheduled Caste	4,572	22.1	1,220	11.4
Scheduled Tribe	2,125	10.3	325	3.0
Other Backward Class	9,213	44.5	5,056	47.1
Others	4,815	23.2	4,139	38.5
** Region**				
North	2,655	12.8	1,293	12.0
Central	4,920	23.7	1,533	14.3
East	5,678	27.4	1,573	14.7
Northeast	691	3.3	226	2.1
West	2,898	14.0	2,662	24.8
South	3,883	18.7	3,451	32.1
Total	20,725	100.0	10,739	100.0

### Percentage of older adults suffering from multimorbidity in India, 2017-18 (Table 2)

Overall, significant urban-rural differences were found in the prevalence of multimorbidity among older adults (difference: 16.3; *p* < 0.001); however it is to be noted that the results are from cross-sectional data (see Table 2). It was higher in urban areas compared to rural counterparts (35.4% vs. 19.1%). Among the obesity-related factors, a significant higher urban-rural difference in the prevalence of multimorbidity was found among older adults who had a high risk waist-hip ratio (difference: 15.9%; *p* < 0.001) followed by those who had high waist circumference (difference: 14.5%; *p* < 0.001). Moreover, among behavior factors, higher urban-rural differences were observed among older adults who did physical activity rarely (difference: 19.8%; *p* < 0.001), followed by those who consumed alcohol (difference: 17%; *p* < 0.001). In the case of individual factors, a significant urban-rural difference was found in the prevalence of multimorbidity among older adults who belonged to other marital status categories followed by those not working. In contrast, this difference was lowest among the oldest-old cohort (difference: 7.3%; *p* < 0.001), followed by those who had higher education (difference: 9.7%; *p* < 0.001). Moreover, a higher percentage of urban-rural difference in the prevalence of multimorbidity was observed among older adults who belonged to the wealthiest families (difference: 24.1% *p* < 0.001).

**Table 2:** Percentage of older adults suffering from multimorbidity in India, 2017-18

Background characteristics	Rural	Urban	Differences	p-value	Cohen’s d
	% (N)	% (N)	%		
**Obesity related factors**					
**Obese/overweight**					
No	16.3 (2911)	28.8 (2063)	12.5	<0.001	-0.35 (-0.38, -0.33)
Yes	36.6 (1048)	48.6 (1737)	11.9	<0.001	-0.19 (-0.24, -0.14)
**High risk waist circumference**					
No	16.2 (2840)	28.0 (1979)	11.8	<0.001	-0.35 (-0.38, -0.32)
Yes	35.1 (1118)	49.6 (1821)	14.5	<0.001	-0.24 (-0.29, -0.2)
**High risk waist-hip ratio**					
No	15.4 (1077)	31.5 (948)	16.1	<0.001	-0.42 (-0.47, -0.38)
Yes	21.0 (2881)	36.9 (2851)	15.9	<0.001	-0.36 (-0.39, -0.33)
**Behavioural factors**					
**Physical activity status**					
Frequent	13.1 (519)	29.0 (466)	15.9	<0.001	-0.45 (-0.51, -0.39)
Rare	12.4(384)	32.2 (261)	19.8	<0.001	-0.42 (-0.49, -0.34)
Never	22.4 (3055)	36.9 (3072)	14.5	<0.001	-0.34 (-0.36, -0.31)
**Tobacco consumption**					
No	20.8 (2358)	37.4 (2947)	16.6	<0.001	-0.38 (-0.41, -0.35)
Yes	17.1 (1601)	29.9 (852)	12.8	<0.001	-0.34 (-0.38, -0.3)
**Alcohol consumption**					
No	19.7 (3440)	35.7 (3400)	16.0	<0.001	-0.37 (-0.4, -0.35)
Yes	15.9 (518)	32.9 (400)	17.0	<0.001	-0.42 (-0.48, -0.35)
**Individual factors**					
**Age**					
Young-old (60-69 years)	17.8 (2156)	34.8 (2183)	17.1	<0.001	-0.39 (-0.43, -0.36)
Old-old (70-79 years)	20.4 (1257)	38.3 (1283)	17.9	<0.001	-0.39 (-0.43, -0.35)
Oldest-old (80+ years)	22.6 (545)	29.8 (333)	7.3	0.012	-0.31 (-0.39, -0.24)
**Sex**					
Male	18.2 (1829)	32.5 (1569)	14.2	<0.001	-0.34 (-0.38, -0.31)
Female	19.9 (2129)	37.8 (2231)	17.9	<0.001	-0.42 (-0.45, -0.39)
**Education**					
Not educated/primary not completed	17.5 (2794)	28.8 (1421)	11.3	<0.001	-0.37 (-0.4, -0.34)
Primary	23.8 (492)	40.8 (617)	17.0	<0.001	-0.32 (-0.38, -0.25)
Secondary	24.0 (476)	42.8 (1110)	18.8	<0.001	-0.24 (-0.3, -0.18)
Higher	28.7 (195)	38.5 (651)	9.7	<0.001	-0.24 (-0.33, -0.15)
**Marital status**					
Currently married	18.7 (2435)	34.3 (2166)	15.6	<0.001	-0.38 (-0.41, -0.35)
Widowed	20.3 (1474)	37.1 (1545)	16.9	<0.001	-0.39 (-0.43, -0.35)
Others	11.4 (48)	33.9 (88)	22.5	0.026	-0.44 (-0.58, -0.3)
**Working status**					
Working	11.1 (810)	24.7 (519)	13.6	<0.001	-0.37 (-0.42, -0.33)
Retired	23.7 (2076)	34.6 (1631)	10.9	<0.001	-0.31 (-0.35, -0.27)
Not working	23.3 (1071)	42.2 (1649)	18.9	<0.001	-0.37 (-0.41, -0.33)
**Household factors**					
**MPCE quintile**					
Poorest	12.9 (572)	25.3 (605)	12.4	<0.001	-0.42 (-0.47, -0.37)
Poorer	16.5 (758)	30.0 (657)	13.5	<0.001	-0.42 (-0.47, -0.36)
Middle	19.1 (833)	30.0 (661)	10.9	<0.001	-0.36 (-0.41, -0.31)
Richer	21.2 (834)	43.3 (916)	22.1	<0.001	-0.41 (-0.47, -0.36)
Richest	28.5 (960)	52.6 (958)	24.1	<0.001	-0.35 (-0.41, -0.3)
**Religion**					
Hindu	18.3 (3164)	35.6 (3026)	17.3	<0.001	-0.39 (-0.42, -0.36)
Muslim	21.5 (434)	33.0 (529)	11.5	<0.001	-0.34 (-0.41, -0.28)
Christian	25.4 (158)	47.9 (129)	22.5	<0.001	-0.37 (-0.45, -0.29)
Others	26.2 (202)	31.2 (115)	5.1	0.008	-0.23 (-0.34, -0.12)
**Caste**					
Scheduled Caste	17.6 (802)	28.6 (349)	11.1	<0.001	-0.32 (-0.38, -0.25)
Scheduled Tribe	10.3 (218)	18.0(58)	7.7	<0.001	-0.5 (-0.57, -0.44)
Other Backward Class	19.9 (1832)	36.3 (1833)	16.4	<0.001	-0.34 (-0.38, -0.3)
Others	23.0 (1106)	37.7 (1559)	14.7	<0.001	-0.3 (-0.34, -0.26)
**Region**					
North	21.3 (565)	32.1 (415)	10.8	<0.001	-0.28 (-0.33, -0.22)
Central	11.3 (557)	22.0 (337)	10.7	0.002	-0.37 (-0.45, -0.3)
East	19.6 (1112)	37.7 (593)	18.1	<0.001	-0.51 (-0.57, -0.45)
Northeast	13.2 (91)	26.9 (60)	13.7	0.105	-0.3 (-0.38, -0.23)
West	22.3 (645)	35.1 (934)	12.8	0.002	-0.31 (-0.37, -0.25)
South	25.4 (986)	42.3 (1458)	16.9	<0.001	-0.26 (-0.31, -0.22)
**Total**	19.1	35.4	16.3	<0.001	

### Estimates from multivariate logistic regression decomposition estimates for urban-rural differentials in the prevalence of multimorbidity among older adults, 2017-18 (Table 3)

Table 3 shows the results of the multivariate decomposition analysis for multimorbidity among older adults by selected variables. The multivariate decomposition logistic regression analysis revealed that about 51% of the overall differences (urban-rural) in the prevalence of multimorbidity among older adults was due to compositional characteristics (endowments). In contrast, the remaining 49% was due to the difference in the effect of characteristics (Coefficient). Among the compositional change factors, obese/overweight, high-risk waist circumference, physical activity status, education, working status, and geographical region significantly affected the change contribution.

The urban-rural differences are explained mainly by geographical region, high-risk waist circumference, working status, obese/overweight, and education. The study found that the regional inequality in the prevalence of multimorbidity among older adult’s accounts for nearly 10% of the explained gap in both groups. Moreover, obese/overweight and high-risk waist circumference were found to narrow the difference in the prevalence of multimorbidity among older adults between urban and rural by 8% and 9.1%, respectively. Finally, work status and education are found to reduce the urban-rural gap in the prevalence of multimorbidity among older adults by 8% and 6%, respectively.

**Table 3:** Multivariate logistic regression decomposition estimates for urban-rural differentials in multimorbidity among older adults in India, 2017-18

Background characteristics	Due to difference in characteristics					Due to difference in coefficients				
	Coef.	SE	*p*-value	Percent		Coef.	SE	*p*-value	Percent	
**Obesity related factors**										
**Obese/overweight**										
No					8.0					-2.4
Yes	0.013	0.002	<0.001	8.0		-0.004	0.002	0.022	-2.4	
**High risk waist circumference**										
No					9.1					-0.3
Yes	0.015	0.002	<0.001	9.1		0.000	0.002	0.809	-0.3	
**High risk waist-hip ratio**										
No					-0.4					-10.5
Yes	-0.001	0.001	0.390	-0.4		-0.017	0.007	0.012	-10.5	
**Behavioural factors**										
**Physical activity status**										
Frequent					3.4					-9.0
Rare	0.000	0.001	0.801	-0.2		0.000	0.003	0.985	-4.5	
Never	0.006	0.002	<0.001	3.6		-0.007	0.008	0.359	-4.5	
**Tobacco consumption**										
No					0.3					-1.1
Yes	0.001	0.002	0.762	0.3		-0.002	0.004	0.673	-1.1	
**Alcohol consumption**										
No					-1.2					2.8
Yes	-0.002	0.001	0.018	-1.2		0.005	0.002	0.060	2.8	
**Individual factors**										
**Age**										
Young-old (60-69 years)					0.4					-0.2
Old-old (70-79 years)	0.000	0.000	<0.001	0.3		0.001	0.003	0.720	0.6	
Oldest-old (80+ years)	0.000	0.000	0.542	0.1		-0.001	0.002	0.448	-0.7	
**Sex**										
Male					0.0					5.1
Female	0.000	0.000	0.789	0.0		0.008	0.006	0.178	5.1	
**Education**										
Not educated/primary not completed					6.1					-3.9
Primary	0.001	0.000	0.002	0.9		-0.002	0.001	0.233	-1.0	
Secondary	0.004	0.001	0.005	2.4		-0.004	0.001	0.004	-2.2	
Higher	0.005	0.002	0.008	2.8		-0.001	0.001	0.048	-0.7	
**Marital status**										
Currently married					-0.2					-0.1
Widowed	0.000	0.000	0.423	0.0		-0.001	0.003	0.814	-0.5	
Others	0.000	0.000	0.310	-0.2		0.001	0.001	0.319	0.3	
**Working status**										
Working					8.1					-10.1
Retired	0.002	0.000	<0.001	1.5		-0.011	0.005	0.023	-6.5	
Not working	0.011	0.002	<0.001	6.7		-0.006	0.003	0.072	-3.6	
**Household factors**										
**MPCE quintile**										
Poorest					-0.3					-2.3
Poorer	0.000	0.000	0.006	0.1		0.000	0.003	0.963	-0.1	
Middle	0.000	0.000	<0.001	-0.2		-0.003	0.003	0.251	-1.9	
Richer	0.000	0.000	<0.001	-0.2		0.001	0.003	0.808	0.4	
Richest	0.000	0.000	<0.001	0.0		-0.001	0.003	0.653	-0.7	
**Religion**										
Hindu					2.2					-2.6
Muslim	0.004	0.001	<0.001	2.6		0.000	0.001	0.866	-0.1	
Christian	-0.001	0.000	0.108	-0.4		-0.002	0.002	0.333	-1.1	
Others	0.000	0.000	0.413	0.1		-0.002	0.001	0.021	-1.4	
**Caste**										
Scheduled Caste					5.2					2.9
Scheduled Tribe	0.004	0.002	0.015	2.7		0.006	0.004	0.079	3.8	
Other Backward Class	0.000	0.000	0.681	0.0		-0.004	0.005	0.457	-2.2	
Others	0.004	0.002	0.093	2.4		0.002	0.003	0.492	1.3	
**Region**										
North					10.4					3.7
Central	0.004	0.001	0.015	2.2		0.004	0.003	0.115	2.6	
East	-0.006	0.001	0.000	-3.4		0.008	0.003	0.010	4.7	
Northeast	0.003	0.001	0.003	1.9		0.000	0.003	0.933	0.1	
West	0.004	0.001	0.003	2.1		-0.004	0.001	0.008	-2.4	
South	0.012	0.002	<0.001	7.6		-0.002	0.003	0.392	-1.3	
Constant						0.119	0.023	<0.001	72.6	72.6
**Total**	0.083	0.004	<0.001	50.9		0.080	0.006	<0.001	49.1	

## Discussion

This study found that the overall prevalence of multimorbidity was lower among older adults in rural areas (19.1% vs. 35.4%) than their urban counterparts. Several previous studies also noted a higher prevalence of multimorbidity among older adults in rural than older adults in urban [13]. The higher risk of multimorbidity in urban areas could be associated with increased prevalence of risk factors such as sedentary urban lifestyle, physical inactivity, and increase in energy and fat intake [26]. Urbanization also contributes to the increase in the prevalence of NCD risk factors [53–55]. Moreover, living in urban areas provides easy access to healthcare facilities, leading to higher health-seeking behaviour (Patel & Chauhan, 2020), leading to prompt diagnosis of NCD, raising the prevalence of multimorbidity in urban areas than in rural areas [56]. Furthermore, increasing nuclear family trends set up in urban areas could also be attributed to a higher risk of multimorbidity among older adults in urban [53].

The study found that obesity was an important factor among older adults experiencing multimorbidity. Results found that those who were obese were more likely to experience multimorbidity in rural and urban areas. Reducing urban-rural inequality in obesity-related factors would decline rural-urban inequalities in multimorbidity by almost 17%. Previous studies in developed as well as developing countries have unanimously agreed on a higher risk of multimorbidity among the obese population than in the non-obese population [27, 44, 57–63]. High waist-hip ratio and high-risk waist circumference were also associated with a high risk of multimorbidity among older adults, as noticed in previous studies [44]. Waist hip ratio and waist circumference were shown to be more sensitive among several anthropometric indices of obesity while screening for multimorbidity [44]. One widely followed hypothesis is that obesity leads to a state of chronic inflammation due to the accumulation of adipose tissue, further leading to stress response; all these events altogether lead to a rise in the incidence of morbidities such as cardiovascular diseases, diabetes, respiratory problems and other such chronic conditions [64, 65]. Moreover, obesity could be a consequence of the presence of multimorbidity, as shown in a prospective study by Nagel et al. (2008), who reported an increase in obesity as the number of morbidities increased [66]. The cross-sectional nature of data limits our understanding of causal inferences, and reverse causation cannot be ruled out in this study also. Obesity is a significant risk factor for chronic diseases, further leading to a higher risk of multimorbidity [61].

Physical inactivity was also noticed as an important risk factor for multimorbidity among older adults. Reducing inequality in physical activity among older adults would decline the urban-rural differential in multimorbidity among them, as shown in this study. Corroborating with previous findings [60, 67–69], this study noted an increased risk of multimorbidity among those who were never involved in physical activity than their counterparts in rural as well as in urban areas. However, a few studies failed to notice any association between multimorbidity and physical inactivity [59, 70]. On the other hand, being physically inactive could be associated with an increased risk of obesity which is well-linked to multimorbidity [59]. Furthermore, engaging in an active lifestyle has proven to be protective against several chronic diseases, such as coronary heart disease [71], diabetes [72, 73], and hypertension [73, 74], which could further be linked to the association between physical activity and multimorbidity.

Education status was another prominent factor infusing urban-rural inequality in the prevalence of multimorbidity among older adults. Results linked higher education to the risk for multimorbidity among older adults. Several previous studies have validated our finding on association between higher education and increased risk of multimorbidity in the Indian context [38]. However, quite a few studies conducted in different settings have noted a high risk of multimorbidity among uneducated older adults than educated older adults [75–77]. In addition, higher education is linked to better health literacy leading to increased consultations with health care providers, therefore increasing the probability of getting diagnosed with more chronic conditions [78, 79]. Another study in the Indian context also noted a positive correlation between higher education and the number of outpatient visits substantiating the claims of a higher risk of multimorbidity among educated people [38].

Confirming the evidence from previous studies [26, 31], this study noted that household wealth was associated with risk of multimorbidity where higher wealth among older adults was linked to a high risk of multimorbidity. This can be attributed to the fact that people from lower socio-economic status are less likely to seek health care and, therefore, less likely to have chronic diseases diagnosed [38]. In addition, higher income is linked to higher treatment-seeking affordability among older adults in India leading to a higher prevalence of multimorbidity [80]. In alignment with previous findings [31, 38, 78, 81], the findings in this study noted a higher prevalence of multimorbidity in female than in male older adults. Therefore, higher treatment-seeking among females at older ages could be attributed to a higher diagnosis of multimorbidity [80].

In agreement with previous studies [26, 82, 83], the prevalence of multimorbidity was higher among non-working groups than in a working group. Working older adults might be involved in some work-related physical activity and may not follow a sedentary lifestyle, which can explain low risk of multimorbidity [56]. The prevalence of multimorbidity was highest among older adults in the Southern region in the country and regions of India highly explained urban-rural inequality in the prevalence of multimorbidity. Previous studies agree with the finding and noticed a high risk of multimorbidity among older adults in residing in Southern part of the country [26, 84]. Kinra et al. (2010) believe that the risk factors associated with multimorbidity are widely prevalent among the South Indian population [84].

### Strength and limitations of the study

The key strength of this study is the use of a recently released nationally representative sample that provides robust estimates of the study variables. However, there are some associated limitations too in this study. This is cross-sectional data and therefore limits the causal understanding. Respondents were asked whether they were diagnosed with any of the chronic diseases by a health professional. This could result in recall bias as some of the respondents might not be able to recollect particular chronic conditions. Furthermore, doctor diagnosis may be biased due to poor quality diagnosis in India [13]. Also, the reporting of multimorbidity was calculated by merging only nine available chronic conditions; there are many more chronic conditions for which data was not available, undermining the prevalence of multimorbidity. The available data only provides the prevalence and determinants related information; therefore limits our understanding of the severity of the diseases/multimorbidity. Furthermore, tobacco and consumption of alcohol has a dose relationship with ill health, therefore findings observed in this study cannot be fully understood due to lack of comprehensive data related to tobacco and alcohol consumption. Also, a significant proportion of population was categorized under the ‘never’ category of physical activity and this seems unrealistic. However, it is worth mentioning that the data on physical activity was collected on a set of domain which could have lead to lower prevalence of physical activity among older adults. Furthermore, household work was not considered as physical activity which could also have some implications. Also, recall bias could not be ruled out in capturing the information related to monthly per capita expenditure. The study estimates could have been affected by the responses from older adults with psychiatric diseases.

## Conclusions

This study provides concrete evidence on the emerging rural-urban inequality in the prevalence of multimorbidity among older adults, highlighting the need for immediate interventions from health planners and policymakers. Specifically, our findings indicate a need for the growing burden of multimorbidity in urban areas to be considered within the framework of several factors predicting high urban-rural inequality in the prevalence of multimorbidity. Results found that controlling obesity-related factors and improving physical activity among older adults would decrease the urban-rural inequality in the prevalence of multimorbidity by almost 20%. Education was another significant predictor of urban-rural inequality in the prevalence of multimorbidity among older adults along with working status. Based on the findings, the study has some valuable suggestions. Although the direction of the relationship between several risk factors and multimorbidity could be debated, there is a need for public health policy to emphasize the importance of reducing the burden of multimorbidity among older adults, specifically in urban areas. There is a need to substantially increase the public sector investment in healthcare to address the multimorbidity among older adults, more so in urban areas, without compromising the needs of older adults in rural areas.

## Data Availability

The datasets generated and/or analysed during the current study are available with the International Institute for Population Sciences, Mumbai, India repository and could be accessed from the following link:https://iipsindia.ac.in/sites/default/files/LASI_DataRequestForm_0.pdf. Those who wish to download the data have to follow the above link. This link leads to a data request form designed by International Institute for Population Sciences. After completing the form, it should be mailed to: datacenter@iips.netfor further processing. After successfully sending the mail, individual will receive the data in a reasonable time.

## Abbreviations

LASI: Longitudinal Ageing Study in India
PSUs: Primary Sampling Units
CEB: Census Enumeration Block
MPCE: Monthly per Capita Expenditure
OBC: Other Backward Class
ST: Scheduled Tribe
SC: Scheduled Caste
NCD: Non-communicable Disease

## References

[1] Wang H (2020). Global age-sex-specific fertility, mortality, healthy life expectancy (HALE), and population estimates in 204 countries and territories, 1950–2019: a comprehensive demographic analysis for the Global Burden of Disease Study 2019. Lancet.

[2] United Nations and Department of Economic and Social Affairs, “World population ageing, 2019 highlights,” NewYork, 2020.

[3] Chauhan S, Arokiasamy P (2018). India’s demographic dividend: state-wise perspective. J Soc Econ Dev.

[4] Patel R, Chauhan S, Chaurasiya D, Kumar S, Paswan B (2019). Role and Impact of Social Capital on Health of Older Adult in India. Indian J Soc Res.

[5] V. Mishra. India’s projected aged population (65+), projected life expectancy at birth and insecurities faced by aged population. Ageing Int. 2019; 1–13.

[6] National Institute on Ageing, “Why Population Aging Matters: A Global Perspective,” National Institutes of Health, 07–6134, 2007. Accessed: May 31, 2021. [Online]. Available: https://journals.plos.org/plosone/article?id=10.1371/journal.pone.0237307

[7] Pearson-Stuttard J, Ezzati M, Gregg EW (2019). “Multimorbidity—a defining challenge for health systems. Lancet Public Health.

[8] Afshar S, Roderick PJ, Kowal P, Dimitrov BD, Hill AG (2015). Multimorbidity and the inequalities of global ageing: a cross-sectional study of 28 countries using the World Health Surveys. BMC Public Health.

[9] Khanam MA, Streatfield PK, Kabir ZN, Qiu C, Cornelius C, Wahlin Å (2011). Prevalence and Patterns of Multimorbidity among Elderly People in Rural Bangladesh: A Cross-sectional Study. J Health Popul Nutr..

[10] Koné AJ, Pefoyo (2015). The increasing burden and complexity of multimorbidity. BMC Public Health.

[11] R. Ofori-Asenso, K. L. Chin, A. J. Curtis, E. Zomer, S. Zoungas, and D. Liew. Recent Patterns of Multimorbidity Among Older Adults in High-Income Countries. Population Health Manag. 2018;22(2);127–137. doi: 10.1089/pop.2018.0069. 10.1089/pop.2018.0069 30096023

[12] Arokiasamy P, Uttamacharya, Jain K (2015). Multi-Morbidity, Functional Limitations, and Self-Rated Health Among Older Adults in India: Cross-Sectional Analysis of LASI Pilot Survey, 2010. SAGE Open.

[13] Pati S (2014). Non communicable disease multimorbidity and associated health care utilization and expenditures in India: cross-sectional study. BMC Health Services Research.

[14] M. van den Akker, F. Buntinx, and J. A. Knottnerus, “Comorbidity or multimorbidity,” *European Journal of General Practice*, vol. 2, no. 2, pp. 65–70, Jan. 1996, doi: 10.3109/13814789609162146.

[15] E. Alonso-Morán, R. Nuño-Solinis, G. Onder, and G. Tonnara Multimorbidity in risk stratification tools to predict negative outcomes in adult population. Eur J Internal Med 2015;26(3):182–189. doi: 10.1016/j.ejim.2015.02.010. 10.1016/j.ejim.2015.02.010 25753935

[16] R. Gijsen, N. Hoeymans, F. G. Schellevis, D. Ruwaard, W. A. Satariano, and G. A. M. van den Bos. Causes and consequences of comorbidity: A review,” Journal of Clinical Epidemiol. 2001;54(7):661–674. doi: 10.1016/S0895-4356(00)00363-2. 10.1016/s0895-4356(00)00363-2 11438406

[17] Salisbury C, Johnson L, Purdy S, Valderas JM, Montgomery AA (2011). Epidemiology and impact of multimorbidity in primary care: a retrospective cohort study. Br J Gen Pract.

[18] F. Castellana et al., “Physical Frailty, Multimorbidity, and All-Cause Mortality in an Older Population From Southern Italy: Results from the Salus in Apulia Study. J Am Med Directors Assoc. 2021; 22(3):598–605. doi: 10.1016/j.jamda.2020.12.026. 10.1016/j.jamda.2020.12.026 33493467

[19] Ge L, Yap CW, Heng BH (2018). Sex differences in associations between multimorbidity and physical function domains among community-dwelling adults in Singapore. PLOS ONE.

[20] U. Kadam, P. Croft, and North Staffordshire GP Consortium Group. Clinical multimorbidity and physical function in older adults: a record and health status linkage study in general practice. Family Practice 2007;24(5)412–419. doi: 10.1093/fampra/cmm049. 10.1093/fampra/cmm049 17698977

[21] Wei MY, Kabeto MU, Langa KM, Mukamal KJ (2018). Multimorbidity and Physical and Cognitive Function: Performance of a New Multimorbidity-Weighted Index. J Gerontol: Series A.

[22] Wei MY, Kabeto MU, Galecki AT, Langa KM (2019). Physical Functioning Decline and Mortality in Older Adults With Multimorbidity: Joint Modeling of Longitudinal and Survival Data. J Gerontol: Series A..

[23] Wei MY, Levine DA, Zahodne LB, Kabeto MU, Langa KM (2020). Multimorbidity and Cognitive Decline Over 14 Years in Older Americans. J Gerontol: Series A.

[24] Li H-W (2021). Quality of Life among Community-Dwelling Middle-Aged and Older Adults: Function Matters More than Multimorbidity. Arch Gerontol Geriatrics.

[25] Salive ME (2013). Multimorbidity in Older Adults. Epidemiol Rev.

[26] Muksor A, Dixit P, Varun MR (2018). Rural-Urban Differentials in NCD Multimorbidity in Adult Population in India: Prevalence and Cost of Care. J Tropical Med Health.

[27] dos Costa CS (2018). Inequalities in multimorbidity among elderly: a population-based study in a city in Southern Brazil. Cad. Saúde Pública.

[28] A. Marengoni, B. Winblad, A. Karp, and L. Fratiglioni. Prevalence of Chronic Diseases and Multimorbidity Among the Elderly Population in Sweden. Am J Public Health 2008; 98(7):1198–1200. doi: 10.2105/AJPH.2007.121137. 10.2105/AJPH.2007.121137 PMC2424077 18511722

[29] Hien H (2014). Prevalence and patterns of multimorbidity among the elderly in Burkina Faso: cross-sectional study. Tropical Med Int Health.

[30] R. Zhang, Y. Lu, L. Shi, S. Zhang, F. Chang. Prevalence and patterns of multimorbidity among the elderly in China: a cross-sectional study using national survey data. BMJ Open 2019;9(8):e024268. doi: 10.1136/bmjopen-2018-024268. 10.1136/bmjopen-2018-024268 PMC6701688 31427309

[31] G. K. Mini and K. R. Thankappan. Pattern, correlates and implications of non-communicable disease multimorbidity among older adults in selected Indian states: a cross-sectional study. BMJ Open 2017;7(3):e013529. doi: 10.1136/bmjopen-2016-013529. 10.1136/bmjopen-2016-013529 PMC5353268 28274966

[32] Banjare P, Pradhan J (2014). “Socio-Economic Inequalities in the Prevalence of Multi-Morbidity among the Rural Elderly in Bargarh District of Odisha (India). PLOS ONE.

[33] Gupta A, Girdhar S, Chaudhary A, Chawla JS, Kaushal P (2016). Patterns of multimorbidity among elderly in an urban area of North India. J Evol Med Dental Sci.

[34] J. S. Kshatri, S. K. Palo, T. Bhoi, S. R. Barik, and S. Pati. Prevalence and Patterns of Multimorbidity Among Rural Elderly: Findings of the AHSETS Study. Front. Public Health 2020;8. doi: 10.3389/fpubh.2020.582663. 10.3389/fpubh.2020.582663 PMC7676903 33251177

[35] S. Pati, S. Swain, M. A. Hussain, S. Kadam, and C. Salisbury. Prevalence, Correlates, and Outcomes of Multimorbidity Among Patients Attending Primary Care in Odisha, India. Ann Fam Med 2015;13(5):446–450. doi: 10.1370/afm.1843. 10.1370/afm.1843 PMC4569452 26371265

[36] S. Pati, S. Swain, J. Metsemakers, J. A. Knottnerus, M. van den Akker. Pattern and severity of multimorbidity among patients attending primary care settings in Odisha, India. PLOS ONE 2017;12(9):e0183966. doi: 10.1371/journal.pone.0183966. 10.1371/journal.pone.0183966 PMC5598947 28910309

[37] Verma V, Mishra N, Mishra N, Mishra N (2019). “A Study on Multi-morbidity among Geriatric Group in a District of Northern India: A Cross Sectional Study. Int J Med Public Health.

[38] Pati S, Swain S, Knottnerus JA, Metsemakers JFM, van den Akker M (2020). Magnitude and determinants of multimorbidity and health care utilization among patients attending public versus private primary care: a cross-sectional study from Odisha, India. Int J Equity Health.

[39] International Institute for Population Sciences (IIPS), NPHCE, MoHFW, Harvard T. H. Chan School of Public Health (HSPH), and The university of Southern California (USC), “Longitudinal Ageing Study in India (LASI) Wave 1,” Mumbai, India, 2020.

[40] Srivastava S, J VJK, Dristhi D, Muhammad T. Interaction of physical activity on the related measures association of obesity- ­ with multimorbidity among older adults: a population- ­ based cross- ­ sectional study in India. BMJ Open, no May. 2021. 10.1136/bmjopen-2021-050245. 10.1136/bmjopen-2021-050245 PMC8144051 34020981

[41] McKenna SP (2011). Measuring patient-reported outcomes: moving beyond misplaced common sense to hard science. BMC Medicine.

[42] Garin N, Study Multi-Country (2016). “Global Multimorbidity Patterns: A Cross-Sectional, Population-Based. J Gerontol: Series A.

[43] WHO, “Obesity and overweight: Fact sheet,” 2016.

[44] Zhang J, Xu L, Li J, Sun L, Qin W (2020). Association between obesity-related anthropometric indices and multimorbidity among older adults in Shandong, China: a cross-sectional study. BMJ Open.

[45] Srivastava S, Gill A (2020). “Untreated morbidity and treatment-seeking behaviour among the elderly in India: Analysis based on National Sample Survey 2004 and 2014. SSM - Population Health.

[46] Srivastava S, Kumar S (2021). “Does socio-economic inequality exist in micro-nutrients supplementation among children aged 6–59 months in India? Evidence from National Family Health Survey 2005–06 and 2015–16. BMC Public Health.

[47] Chauhan S, Sekher TV, Kumar P, Srivastava S, Patel R (2020). “Prevalence, determinants and socio-economic inequality of early marriage among men in India. Children Youth Serv Review.

[48] Fan C, Wang L, Wei L (2017). Comparing Two Tests for Two Rates. Am Statistician.

[49] D. A. Powers, H. Yoshioka, and M.-S. Yun. Mvdcmp: Multivariate Decomposition for Nonlinear Response Models. Stata J 2011:11(4): 556–576. doi: 10.1177/1536867X1201100404.

[50] Tiruneh SA, Lakew AM, Yigizaw ST, Sisay MM, Tessema ZT (2020). Trends and determinants of home delivery in Ethiopia: further multivariate decomposition analysis of 2005–2016 Ethiopian Demographic Health Surveys. BMJ Open.

[51] A. Debie, A. M. Lakew, K. S. Tamirat, G. Amare, G. A. Tesema. Complete vaccination service utilization inequalities among children aged 12–23 months in Ethiopia: a multivariate decomposition analyses. Int J Equity Health 2020 19(1):65. doi: 10.1186/s12939-020-01166-8. 10.1186/s12939-020-01166-8 PMC7218567 32398089

[52] StataCorp L (2015). Stata statistical software (version release 14). College Station, TX: Author.

[53] Cantarero-Prieto D, Pascual-Sáez M, Blázquez-Fernández C (2018). Social isolation and multiple chronic diseases after age 50: A European macro-regional analysis. PLOS ONE.

[54] P. S. Shetty. Nutrition transition in India. Public Health Nutr, 2002;5(1A):175–182. doi: 10.1079/PHN2001291. 10.1079/PHN2001291 12027282

[55] Yadav K, Krishnan A (2008). “Changing patterns of diet, physical activity and obesity among urban, rural and slum populations in north India. Obesity Reviews.

[56] Srivastava S, Anwar T, Patel R, Chauhan S (2020). Dynamics of chronic diseases in metro and non-metro regions of India: evidence from India Human Development Survey I and II. Int J.

[57] Agborsangaya CB, Lau D, Lahtinen M, Cooke T, Johnson JA (2012). Multimorbidity prevalence and patterns across socioeconomic determinants: a cross-sectional survey. BMC Public Health.

[58] Agrawal S, Agrawal PK (2016). Association Between Body Mass index and Prevalence of Multimorbidity in Low-and Middle-income Countries: A Cross-Sectional Study. Int J Med Public Health.

[59] V. de S. Santos Machado, A. L. R. Valadares, L. H. Costa-Paiva, M. J. Osis, M. H. Sousa, and A. M. Pinto-Neto, “Aging, obesity, and multimorbidity in women 50 years or older: a population-based study,” *Menopause*, vol. 20, no. 8, pp. 818–824, Aug. 2013, doi: 10.1097/GME.0b013e31827fdd8c. 10.1097/GME.0b013e31827fdd8c 23549445

[60] de Souza Santos Machado V, Valadares ALR, da Costa-Paiva LS, Moraes SS, Pinto-Neto AM (2012). Multimorbidity and associated factors in Brazilian women aged 40 to 65 years: a population-based study. Menopause.

[61] C. Diederichs, K. Berger, and D. B. Bartels. The Measurement of Multiple Chronic Diseases—A Systematic Review on Existing Multimorbidity Indices. J Gerontol: Series A, 2011; 66A(3)301–311. doi: 10.1093/gerona/glq208. 10.1093/gerona/glq208 21112963

[62] H.-J. Dong, M. Unosson, E. Wressle, and J. Marcusson. Health Consequences Associated with Being Overweight or Obese: A Swedish Population-Based Study of 85-Year-Olds. J Am Geriatrics Soc 2012; 60(2):243–250, 2012, doi: 10.1111/j.1532-5415.2011.03827.x. 10.1111/j.1532-5415.2011.03827.x 22283806

[63] Jovic D, Marinkovic J, Vukovic D (2016). Association between body mass index and prevalence of multimorbidity: a cross-sectional study. Public Health.

[64] Cancello R, Clément K (2006). “Review article: Is obesity an inflammatory illness? Role of low-grade inflammation and macrophage infiltration in human white adipose tissue. BJOG.

[65] Guri AJ, Josep BR (2011). Systemic Effects of White Adipose Tissue Dysregulation and Obesity-Related Inflammation. Obesity.

[66] G. Nagel, R. Peter, S. Braig, S. Hermann, S. Rohrmann, and J. Linseisen, “The impact of education on risk factors and the occurrence of multimorbidity in the EPIC-Heidelberg cohort,” *BMC Public Health*, vol. 8, no. 1, p. 384, Nov. 2008, doi: 10.1186/1471-2458-8-384. 10.1186/1471-2458-8-384 PMC2614432 19014444

[67] C. S. Autenrieth et al. Physical activity is inversely associated with multimorbidity in elderly men: Results from the KORA-Age Augsburg Study. Preventive Med. 2013 57(1):17–19. doi: 10.1016/j.ypmed.2013.02.014. 10.1016/j.ypmed.2013.02.014 23485795

[68] Dhalwani NN (2016). Long terms trends of multimorbidity and association with physical activity in older English population. Int J Behav Nutr Phys Act.

[69] M. S. Kaplan, J. T. Newsom, B. H. McFarland, and L. Lu. Demographic and psychosocial correlates of physical activity in late life. Am J Preventive Med 2001 ;21(4):306–312. doi: 10.1016/S0749-3797(01)00364-6. 10.1016/s0749-3797(01)00364-6 11701302

[70] Hudon C, Soubhi H, Fortin M (2008). Relationship between multimorbidity and physical activity: Secondary analysis from the Quebec health survey. BMC Public Health.

[71] Hakim AA (1999). Effects of Walking on Coronary Heart Disease in Elderly Men. Circulation.

[72] G. Hu et al. Occupational, commuting, and leisure-time physical activity in relation to risk for Type 2 diabetes in middle-aged Finnish men and women. Diabetologia 2003; 46(no. 3); 322–329. doi: 10.1007/s00125-003-1031-x. 10.1007/s00125-003-1031-x 12687329

[73] Kapil U (2018). Prevalence of hypertension, diabetes, and associated risk factors among geriatric population living in a high-altitude region of rural Uttarakhand, India. J Family Med Prim Care.

[74] Alam M, Soni G, Jain K, Verma S, Panda P (2015). Prevalence and determinants of hypertension in elderly population of Raipur city, Chhattisgarh. Int J Res Med Sci.

[75] V. Johnson-Lawrence, A. Zajacova, and R. Sneed. Education, race/ethnicity, and multimorbidity among adults aged 30–64 in the National Health Interview Survey. SSM - Population Health. 2017; 3:366–372. doi: 10.1016/j.ssmph.2017.03.007. 10.1016/j.ssmph.2017.03.007 PMC5769041 29349230

[76] Pathirana TI, Jackson CA (2018). Socioeconomic status and multimorbidity: a systematic review and meta-analysis. Aust New Zealand J Public Health.

[77] Schiøtz ML, Stockmarr A, Høst D, Glümer C, Frølich A (2017). Social disparities in the prevalence of multimorbidity – A register-based population study. BMC Public Health.

[78] S. Pati et al. Prevalence and outcomes of multimorbidity in South Asia: a systematic review. BMJ Open. 2015;5(no. 10):e007235. doi: 10.1136/bmjopen-2014-007235. 10.1136/bmjopen-2014-007235 PMC4606435 26446164

[79] S. M. Smith, H. Soubhi, M. Fortin, C. Hudon, and T. O’Dowd. Managing patients with multimorbidity: systematic review of interventions in primary care and community settings. BMJ 2012;345:e5205. doi: 10.1136/bmj.e5205. 10.1136/bmj.e5205 PMC3432635 22945950

[80] Patel R, Chauhan S (2020). Gender differential in health care utilisation in India. Clin Epidemiol Global Health.

[81] Audinarayana N (2017). Gender Perspectives of Multi-morbidity among Elderly and It’s Determinants in an Urban Setting of Tamil Nadu. Indian J Gerontol.

[82] Björklund O, Söderlund M, Nyström L, Häggström E (2015). Unemployment and Health: Experiences Narrated by Young Finnish Men. Am J Mens Health.

[83] L. Picco et al. Economic burden of multimorbidity among older adults: impact on healthcare and societal costs. BMC Health Serv Res 2016; 16 doi: 10.1186/s12913-016-1421-7. 10.1186/s12913-016-1421-7 PMC4862090 27160080

[84] S. Kinra et al. Sociodemographic patterning of non-communicable disease risk factors in rural India: a cross sectional study. BMJ, 2010; 341 no. sep27 1, c4974–c4974. doi: 10.1136/bmj.c4974. 10.1136/bmj.c4974 PMC2946988 20876148

